# Prediction of Right Heart Failure After Left Ventricular Assist Device: Opening Pandora’s Box

**DOI:** 10.31083/RCM40249

**Published:** 2025-12-24

**Authors:** Claudia Maria Loardi, Marco Zanobini

**Affiliations:** ^1^Department of Cardiac Surgery, Tours University Hospital, 37044 Tours, France; ^2^Department of Cardiovascular Surgery, Centro Cardiologico Monzino IRCCS, 20138 Milan, Italy

**Keywords:** left ventricular assist device, end-stage heart failure, right ventricular function, echocardiography

## Abstract

Continuous-flow left ventricular assist devices (LVADs) represent a leading option in the treatment of end-stage heart failure (HF), provided that right ventricular (RV) contractile function is sufficiently preserved to ensure cardiac output after LVAD implantation. In this context, evaluating the RV before surgery is crucial, as the onset of early right heart failure (RHF) following LVAD placement is linked to increased mortality and morbidity. Unfortunately, the contractile performance of the RV is a difficult issue to evaluate and requires a multimodal approach based on the application of multiple diagnostic tools, including clinical assessment, echocardiography, right heart catheterization (RHC), and risk models, all of which have variable predictive power in the currently available literature. Pre-implantation RV assessment is even more challenging and misleading in patients with hemodynamic instability under extracorporeal membrane oxygenation (ECMO) support, a situation characterized by complete right heart unloading, which renders most assessment techniques unreliable. The present paper proposes a simple and comprehensive preoperative appraisal strategy for the RV, which is adapted to the clinical status (critical or more stable) of the patient, based on a review of the advantages and limitations of each diagnostic modality and derived parameters.

## 1. Introduction

Heart failure (HF) is a multifaceted and life-threatening syndrome affecting more than 64 million people worldwide, characterised by significant morbidity and mortality, poor functional capacity and quality of life, and high costs [1]. Continuous-flow left ventricular assist devices (LVADs) are increasingly used as bridge-to-transplant or destination therapy in patients with advanced HF [2]. As their function is to support only the left ventricle, the main prerequisite for successful implantation and correct functioning is the presence of a sufficiently well-contracting right ventricle (RV), able to ensure and receive a restored left output. However, the problem lies in the word “sufficient”: it is extremely rare for an end-stage HF condition requiring mechanical assistance to be secondary to an isolated left ventricular dysfunction, either because the patient suffers from a primitive biventricular disease, or because a long-standing left ventricular dysfunction has caused damage to the pulmonary circulation leading to the involvement and deterioration of RV contraction. This process may or may not be partially reversible, the RV may have a contractile reserve, or even in the case of an imperfect RV, its performance may be sufficient to allow a satisfactory patient outcome under LVAD. Furthermore, the RV is a dynamic and adaptable structure that may take several weeks to regain contractile performance. For these reasons, the definition and pre-implantation identification of a “sufficient” RV is difficult and depends on many factors.

The development of right heart failure (RHF) occurs postoperatively in approximately 10–40% of LVAD patients and is associated with a significant increase in perioperative mortality and major complications, including prolonged hospital stay, gastrointestinal bleeding, multiorgan failure and thromboembolism [2]. In addition, another element must be taken into account: a planned temporary RV support or an “emblé” shift towards a definitive biventricular assistance allows a better outcome than a perioperative failure of a left support [3]. It is therefore easy to understand why the preoperative assessment of the RV has attracted the attention of clinicians and has become the real question to be answered in the decision tree when screening a patient for an LVAD.

The aim of the present review is to summarise the current knowledge and the pros and cons of all available tools that may help in the comprehensive assessment of the RV before LVAD implantation in order to predict the occurrence of RHF.

## 2. An Overview of the Diagnostic Tools for the RV

The RV has long been considered a dispensable chamber that does not contribute significantly to overall cardiac function. However, studies published in recent years have shown that RV function is an important independent predictor of morbidity and mortality in several cardiac diseases, including ischaemic cardiomyopathies, heart failure and pulmonary hypertension [4].

In the clinical setting, due to the complex shape, geometry and location of the RV within the thorax, multimodality assessment is currently recommended to obtain a complete evaluation of its structure, size, function and mechanics, overcoming the respective advantages and limitations of each diagnostic tool. More specifically, 2-dimensional and 3-dimensional echocardiography are the first choice imaging modalities due to their high availability. After the initial echocardiographic assessment, cardiac magnetic resonance (CMR) should be performed as a second-line imaging technique because it allows clear visualisation of anatomy, tissue characterisation, quantifying function, and calculating flows. Computed tomography (CT) should be considered in patients with specific contraindications to CMR and is not particularly reliable for volume estimation.

In conclusion, multimodality in the assessment of RV function and deformation allows us to compare the same parameters between different techniques; when an accurate evaluation is required, such as in patients undergoing cardiac surgery, the use of ≥2 imaging modalities is recommended, and one of them should be CMR [5].

## 3. Definition and Types (Early, Late) of RV Dysfunction 

The INTERMACS definition of RHF is “symptoms or findings of persistent RHF characterised by both [6]:

- Documentation of elevated central venous pressure (>16 mmHg) by direct measurement, by significantly dilated inferior vena cava in the absence of inspiratory variation by echocardiography, or by clinical evidence of increased jugular venous distension.

- Manifestations of increased central venous pressure, clinical findings of peripheral oedema, ascites or hepatomegaly, or laboratory evidence of worsening hepatic (bilirubine >2.0 mg/dL) or renal (creatinine >2.0 mg/dL) dysfunction.”

RHF following LVAD implantation most commonly occurs in the early postoperative period (early acute RHF), requiring the implantation of a temporary or permanent right ventricular assist device (RVAD) alongside LVAD placement. A second category of post-LVAD RHF is early post-implant RHF, which is defined as the need for an RVAD within 30 days of LVAD implantation, failure to wean from inotropic or vasopressor support or inhaled nitric oxide within 14 days, or death occurring within 14 days of LVAD implantation in patients who have not received an RVAD but remain on inotropes or vasopressors at the time of death due to documented RHF. Finally, late RHF is defined as the need for an RVAD to be implanted, or as hospitalisation for at least 72 hours due to RHF criteria requiring intravenous diuretics or inotropic support, occurring more than 30 days after LVAD implantation [7].

## 4. Physiology of Early Right Ventricular Dysfunction After LVAD 

In LVAD recipients, the incidence of acute-early RHF ranges from 9% to 44% and could be considered as a two-hit phenomenon: the first hit (preoperative) is the degree of RV impairment that the patient has prior to LVAD implantation, and the second hit (intra- and postoperative) is related to surgical and anatomical factors that negatively affect RV contractility after the institution of LVAD support [8]. These latter factors include loss of pericardial restraint, the effect of left ventricular unloading on septal contractility, ventricular dissynchrony, particularly with increasing LVAD speed, an increase in RV preload, and changes in tricuspid valve competence [9].

Postoperative vasoplegia is another clinical non-RV factor in the pathophysiology of early RHF. This phenomenon is influenced by the inflammatory response to cardiopulmonary bypass, transfusion of blood products and the presence of pre-existing liver disease [10]. From a pathophysiological perspective, to overcome the postoperative reductions in systemic vascular resistance, robust cardiac output from both the left and right ventricles is required to maintain acceptable mean arterial pressures in the early postoperative period. However, while the left ventricle is supported by the LVAD, the RV remains unassisted and, if dysfunctional, is often unable to meet the haemodynamic and metabolic demands.

In the complex interplay between these two factors, the baseline condition plays a predominant role, because it is easy to imagine that the combination of careful management of peri- and post-operative variables together with careful augmentation of the LVAD speed may be able to prevent early RHF in the case of sufficiently preserved RV function. That is the reason why the clinician’s attention should be focused on the evaluation of the pre-implant status of the RV, analysed with all available tools.

## 5. Prediction of Right Heart Failure Following LVAD

### 5.1 Clinical Factors 

Careful clinical assessment for objective signs of advanced RHF is the first and simplest step in pre-LVAD implantation. Long-standing distended jugular veins and peripheral oedema are indicators of RHF that require special attention and possible optimisation before proceeding with the procedure. Potapov *et al*. [11] reported that visible ascites and discolouration of the skin of the legs due to haemosiderosis already progressing above the knees are supportive of long-standing RHF with poor chances of reversibility. These clinical findings make the option of biventricular mechanical support inevitable.

Furthermore, the background of HF seems to play a significant role in the incidence of RV dysfunction after LVAD implantation. Patients with non-ischemic HF have been shown to have an increased risk (absolute value of 5.1%) of early RHF compared to patients with ischemic HF, although the long-term impact of these diseases on late RV function is still unclear [12].

Other markers of pre-implantation patient severity are associated with a higher risk of RHF, including preoperative ventilator support, multiple inotropes, extracorporeal membrane oxygenator (ECMO) support and non-elective intra-aortic balloon pump (IABP) [13].

### 5.2 Biochemical Values

Abnormal biochemical values including serum creatinine >1.9–2.0 mg/dL, blood urea nitrogen >44.5 mg/dL and total bilirubin >2.0 mg/dL [14, 15, 16] are predictors of RV impairment after left ventricular support. In addition, a meta-analysis [17] found that patients on continuous renal replacement therapy or on mechanical ventilation at the time of LVAD placement were more likely to have persistent RHF. Similarly, a pro-inflammatory milieu (with a PCT cut-off of 0.09 ng/mL) and significantly low haemoglobin levels [18] have been shown to be detrimental, although they require further validation, since they have been tested on a limited sample size of thirteen patients who developed RHF after LVAD placement.

Such findings are likely to reflect the presence of RHF-related end-organ dysfunction, anaemia or subclinical infection, or simply an inflammatory status (which is very common in patients receiving temporary support with Impella (Abiomed, Inc., Danvers, MA, USA) devices or ECMO), which may or may not be fully reversible after LVAD implantation, thereby complicating postoperative management.

### 5.3 Echocardiography

Echocardiography is the easiest diagnostic modality to perform and repeat. It can provide insight into RV morphology, function and associated structural or functional abnormalities and their dynamic changes that may lead to the development of RHF.

Several echocardiographic parameters have been tested in clinical trials for their prognostic power for early RV dysfunction in the context of LVAD implantation.

Starting with standard two-dimensional transthoracic echocardiography (2D-TTE), the simple presence of a dilated tricuspid annulus has recently been recognised as an independent predictor of late RV dysfunction after LVAD placement [19], while the impact of the RV/LV ratio is far from unanimous [20, 21]: despite using the same measurement technique, Kukucka found a positive correlation between the ratio and RHF development. However, as only 13% of patients in their cohort suffered from serious post-LVAD RHF, their conclusion is less reliable.

A recent meta-analysis by Chriqui *et al*. [22] in a total of 1561 patients showed that RV fractional area change (FAC) and RV global longitudinal strain (RVGLS) are likely to be strong predictors of RHF after LVAD implantation; moreover, in contrast to previous single studies including a limited number of patients in different haemodynamic conditions [21, 23], tricuspid annular plane systolic excursion (TAPSE) emerged as a reliable factor in predicting RHF. The already existing uncertainty about the real role of TAPSE is that it describes apex-to-base shortening and thus reflects only one plane of RV contraction; moreover, it is strongly influenced by changes in volume and preload and increases in response to dobutamine infusion [24]; perhaps its combination with other values represents a suggestive way to strengthen its significance, as suggested by Benedetto *et al*. [24], whose systematic review describes a trend of statistical correlation between TAPSE, FAC and RVGLS with RHF event after LVAD placement. Peak systolic velocity of the tricuspid annulus by pulsed-wave tissue Doppler imaging has been observed to provide prognostic information for RHF after LVAD implantation, with cut-off values ranging from 8.0 to 8.8 cm/s, although data are conflicting [25]. An alternative to overcome the limitations of TAPSE and FAC is the use of 2DTTE with electronic plane rotation, which allows multi-plane quantification of the RV wall for TAPSE and tricuspid annular peak systolic velocity (RV-S’) measurements, which may reveal differences in regional RV wall function [26].

Speckle-tracking echocardiography has been used in LVAD recipients to assess its ability to predict early postoperative RHF: more specifically, lower values of RVGLS [27] and RV free wall strain (with a cut-off value ≤–15.5%, [15]) are associated with an increased risk of developing RV failure; interestingly, only TTE-derived strain parameters, but not those derived from transesophageal echocardiography (TOE), were found to be significant [28]. Similar conclusions were drawn in the cohort of Aymami [29], who found that the quantitative evaluation of both RV size and function with the association of RV end-diastolic area index and free-wall longitudinal strain was a prognostic marker for RHF after LVAD implantation. Following the conclusions of two other retrospective studies [18, 30], RV free wall strain rather than global longitudinal strain appears to play a more important role in influencing post-LVAD RHF.

Based on the same optic of combining both morphological and functional aspects of the RV to strengthen the predictive power of TTE in the selection of adequate candidates for LVAD, a kind of hybrid echocardiographic index represented by the pressure-dimension one (calculated by dividing the systolic pulmonary artery pressure by the square of the RV minor diameter) has been identified as a possible new marker of an overburdened RV more prone to experience early failure after left assistance [31].

Another interesting measure is represented by the interventricular-septal output (ISO), calculated as (systolic interventricular septum – diastolic interventricular septum) × heart rate, whose decrease by 25% or more from pre-implant to hospital discharge seems to be associated with RHF [32]. Such a conclusion confirms the strong influence of mechanical ventricular interdependence on RV function and highlights the paramount role of the septal wall.

Concerning TOE, the application of three-dimensional technology seems promising, as 3D-RV ejection fraction and free wall strain have shown a high discriminatory ability (0.876 and 0.914, respectively) in the detection of RHF after LVAD, although tested in a small cohort of patients [33]. 3D measurement of RV end-diastolic volume index (both on TTE and TOE) and end-systolic volume on TTE (with a cut-off of >47 mm/m^2^ identified by Kiernan) are associated with the postoperative development of RHF [34, 35]. However, 3D RV ejection fraction showed no predictive value in Kiernan’s cohort.

Silverton *et al*. [36] and Alfirevic *et al*. [19] have analysed the impact of several intraoperative TOE-derived parameters on the subsequent occurrence of RHF: interestingly, and in contrast to the same indices calculated at TTE, TAPSE, RV-S’, tricuspid annular displacement and RV strain failed to predict RV impairment, whereas reduced post-bypass FAC was significantly associated with RVF but with poor discrimination. A possible explanation for such conflicting results may be that RV function is highly dependent on haemodynamic conditions and filling; thus, as the patient is under general anaesthesia during intraoperative TOE examination, the performance of RV indices may be confounding.

To overcome the problem of RV load dependence, two TTE-derived dynamic measures, represented by the RV load adaptation index (LAI_RV_) and the RV load-corrected peak global systolic longitudinal strain rate (PSSrL × ΔP_RV-RA_), appear very promising, showing very high predictive values (≥87%) for both RHF and freedom from RHF after LVAD placement for their composite use [37].

In conclusion, the echocardiographic assessment of a patient candidate for LVAD support is far from simple and often requires the association of standard 2D basal values integrated with 3D reconstructions, TOE incidences and speckle-tracking technology.

Although TAPSE, FAC and RV free wall strain appear to correlate well with post-implantation RHF development, the dynamic variables represented by LAI and PSSrL provide greater reliability by incorporating the patient’s overall loading status, which has an important impact on RV characteristics and performance (Table 1).

**Table 1.** **Performance of echocardiographic right ventricular parameters to predict right heart failure following left ventricular device implantation (summary of current evidence)**.

Echocardiographic parameter	Cut-off value	Predictive power
Dilated tricuspid annulus	40 mm or 21 mm/m^2^	Good
RV/LV ratio	>0.72	Uncertain
FAC	≤31%	Good
Peak systolic tricuspid annular velocity	4.4 or 8 cm/s	Uncertain
TAPSE	>16 mm	Uncertain
RVEF		Weak
RVGLS	Nor recognized	Strong
RV free wall strain	≤–15.5%	Strong
Pressure/dimension index	>3.62 mmHg/cm^2^	Strong
Interventricular septum output	Decrease ≤25%	Strong
3D RVEF		Uncertain
3D free wall strain		Strong
3D RV end-diastolic volume index	>62 or >84 mL/m^2^	Strong
3D RV end-systolic volume	>47 mL/m^2^	Strong
Intraoperative TOE parameters		Weak
LAIRV + PSSrL × ΔP_RV-RA_		Very strong

RV, right ventricle; FAC, fractional area change; LAI_RV_ + PSSrL × ΔP_RV-RA_, right ventricular load adaptation index + RV load-corrected peak global systolic longitudinal strain rate; LV, left ventricle; RVEF, right ventricular ejection fraction; RVGLS, right ventricular global longitudinal strain; TAPSE, tricuspid annular plane systolic excursion; TOE, transoesophageal echocardiography; 3D, three-dimensional.

### 5.4 CT

Currently, CT is not widely used in the preoperative evaluation of the RV in patients scheduled for LVAD implantation, mainly due to the potential nephrotoxicity of the contrast and the need for a stable cardiac rhythm with a low heart rate for image acquisition [4].

Nevertheless, contrast-enhanced electrocardiogram-gated CT angiography (cine-CT)—in which multiple CT images are acquired over the entire cardiac cycle—can be used to perform RV volumetric assessment, and values have been validated against CMR. Scott *et al*. [38] decided to apply this diagnostic tool in a small cohort of patients on the LVAD waiting list, and showed that CT-derived RV end-systolic and end-diastolic volume indices were the strongest predictors of RHF compared with demographic, echocardiographic and right heart catheterisation (RHC) data, with areas under the receiver operating curve of 0.79 and 0.76, respectively. Although these results are encouraging, the very limited number (7) of patients experiencing RHF and the difficulty of performing cardiac CT in unstable or assisted patients make it difficult to apply this method on a large scale.

### 5.5 CMR and Radionuclide Angiography

CMR is the current gold standard for assessing RV volumes and function. However, CMR remains a relatively expensive diagnostic tool that is not readily available for bedside use. It is also contraindicated in patients with metallic implants (most patients with end-stage HF have a defibrillator or multi-chamber pacemaker) and is limited to haemodynamically stable conditions. For this reason, 3D echocardiography is becoming widely used in clinical practice due to its low cost and availability, although its validation against CMR in quantifying RV volumes and function is still limited, with poor data on inter-observer variability; this issue warrants further investigation [39].

To our knowledge, only one study [40] in the available literature has attempted to compare the accuracy of RV ejection fraction assessment prior to assist device placement using first pass radionuclide angiography: the results were unsatisfactory, with a moderate correlation between angiographic values and reference standard parameters calculated by CMR and echocardiography.

### 5.6 Invasive Haemodynamics 

RHC before LVAD implantation is a standard diagnostic procedure to assess RV function, and several invasive haemodynamic variables have been identified as possible markers of early postoperative RHF development. These include an elevated right atrial pressure (RAP, >15 mmHg), reflecting increased RV preload [18], a low mean pulmonary artery pressure with impaired RV systolic function, an RAP/PCWP ratio >0.63, as well as increased pulmonary vascular resistance (PVR) and an elevated (>0.55) cardiac filling pressure ratio (mean right atrial to mean pulmonary capillary wedge pressure ratio) which showed an acceptable area under the curve value of 68% [16, 37, 41, 42]. The isolated finding of reduced pulmonary capillary wedge pressure (PCWP) also appears to be a significant predictor of postoperative RHF within one year [27], probably related to the predominance of precapillary pulmonary hypertension leading to a permanent increase in pulmonary resistance. Interestingly, both higher pulmonary arterial pressure and pulmonary arterial pulse pressure seem to protect against RV failure [13], probably because they act as a training condition for the RV, which is forced to muscularise and push against an important obstacle.

Recently, clinicians’ attention has focused on the potential role of the pulmonary artery pulsatility index (PAPi), a simple haemodynamic calculation [(systolic PA-dyastolic PA)/mean right atrial pressure] initially studied to predict RV dysfunction in adults after acute inferior myocardial infarction [43]. In the context of paediatric LVAD placement, the need for inotropes/pulmonary vasodilators in the postoperative period can be predicted by pre-implantation RV intrinsic contractile reserve as assessed by PAPi rather than by markers of RV afterload [44]. In the adult population, the association between PAPi and post-assistance RHF seems to exist, although not unanimously: on the one hand, Kiernan *et al*. [45] suggested that patients with a PAPi less than 1.85 have an increased risk of RHF after LVAD; similarly, another study has demonstrated an association of low preoperative PAPi (<2.84) with a higher risk of early mortality after the left device placement without affecting 2-year outcomes in survivors [46], as well as a more robust predictive power for RHF when postoperative PAPi (with a cut-off value <1.56) is included in the Michigan score [47]. On the contrary, Alfirevic *et al*. [19] did not find any reliability of intraoperative PAPi. Again, it can be argued that the measurement of haemodynamic variables concerning the RV under general anaesthesia cannot be compared to a preoperative RHC under “physiological” conditions. It is impossible to detect the reason why different studies identified such a large discrepancy in the discriminating threshold of PAPi, even though the value of 1.85 seems to be the most reliable, as it emerges from a large cohort of around 10,000 LVAD patients [45].

Other parameters derived from RHC, such as RV stroke work index (RVSWi) of >300 mmHg × mL/m^2^ preoperatively, minimise the risk of perioperative RV assist, thus confirming it as a reliable predictor of RHF after LVAD with an area under the curve of 63% [48]. The important role of RVSWi is confirmed by Gumus *et al*. [15], although they identified a different cut-off value (<400 mmHg × mL/m^2^) to anticipate an adverse RV outcome.

Table 2 summarises the different RHC variables and their predictive power.

**Table 2.** **Performance of right heart catheterization parameters to predict right heart failure following left ventricular device implantation (summary of current evidence)**.

Variable	Cut-off value	Predictive power
RAP	<12 mmHg	Good
Mean PAP + impaired RV function	Not recognized	Good
PVR	Not recognized	Weak
Filling pressure ratio	<0.55	Good
PCWP	Not recognized	Good
RAP/PCWP	<0.63	Good
PAPi	<1.85 or <2.84	Debated
RVSWi	>300 mmHg × mL/m^2^ or >400 mmHg × mL/m^2^	Strong

PAP, pulmonary artery pressure; PAPi, pulmonary artery pulsatility index; PCWP, pulmonary capillary wedge pressure; PVR, pulmonary vascular resistance; RAP, right atrial pressure; RVSWi, right ventricular stroke work index.

### 5.7 Risk Scores 

Given the complexity of global RV assessment, risk scoring studies have emerged since the 2000s to better predict RV behaviour after implantation. By incorporating clinical, laboratory and haemodynamic factors, they attempt to take into account the full complexity of end-stage HF patients, focusing on the degree of RV involvement in the process.

The Michigan right ventricular failure risk score developed by the authors is the first model for preoperative risk stratification of RV failure in LVAD candidates [14]. The score is composed of four variables: preoperative vasopressor use (add 4 points), creatinine >2.3 mg/dL (add 3 points), bilirubin >2 mg/dL (add 2.5 points) and aspartate aminotransferase >80 UI/dL (add 2 points); scores above 5.5 are associated with a higher risk of RV failure after LVAD implantation. In the original validation study, this model showed very effective discriminatory power (area under the curve - AUC of 0.73), but a more recent cohort analysis [19] was less satisfactory, similar to PAPi and other echocardiographic variables.

In the same year, the Penn RVAD risk score was developed by Fitzpatrick *et al*. [49]. This study established a risk model by showing that a low preoperative cardiac index and RVSWi, severe pre-LVAD RV dysfunction, high creatinine level, previous cardiac surgery and hypotension increased the risk of RHF after LVAD implantation. Analysis of this risk score showed that patients with a low score were predicted to have successful LVAD support, whereas patients with a high score were likely to have biventricular assist device (BiVAD) placement.

The UTAH score, elaborated with multivariate regression analysis, identified the need for preimplantation intra-aortic balloon counterpulsation, increased PVR and destination therapy as significant predictors of RHF [50]. With a remarkable AUC of 0.743, the UTAH score is among the more powerful risk models for both RHF and reduced survival at days 30, 180 and 365 after LVAD implantation.

In 2010, Kormos *et al*. [48] evaluated the incidence, risk factors, and impact on outcomes of RHF in patients implanted with a HeartMate II (Abbott, Plymouth, MN, USA), the so-called Kormos score. Multivariate analysis showed that a RAP/PCWP greater than 0.63, preoperative ventilatory support and a blood urea nitrogen level greater than 39 mg/dL were independent predictors of RHF, although the rates of need for temporary RV assist devices were low compared with previous results with pulsatile LVADs, supporting the use of new-generation continuous-flow devices.

An alternative high performance risk model (AUC 0.74) is the CRITT one [51], which includes RAP >15 mmHg, severe RV dysfunction, preoperative intubation, severe tricuspid regurgitation (TR) and tachycardia. By assigning a score of 1 to each variable, Atluri *et al*. [51] showed that 80% of patients with a score of 4 or higher required BiVAD. A particularly appreciated feature of the CRITT model is that it can be easily calculated at the bedside.

More recently, Raina *et al*. [23] and Aissaoui *et al*. [52] proposed two risk models (the TTE and ARVADE scores, respectively) based mainly on echocardiographic parameters, concluding that the combination of different echocardiographic variables could reflect global left ventricular systolic and diastolic dysfunction and RV congestion, thus estimating suitability for LVAD implantation with good reliability.

In 2018, a simple and easy-to-remember risk stratification tool (the ALMA score) [53] was introduced to determine whether an isolated continuous-flow LVAD could be tolerated. A five-point risk score was developed based on clinical variables identified by multivariate logistic regression analysis as follows: the destination therapy intention, PAPi <2, RVSWi <300 mL/m^2^, RV/left ventricle ratio >0.75, and model for end-stage liver disease excluding international normalized ratio (MELD-XI) score >17. BiVAD was recommended for patients with a score of 4 or 5.

Finally, the EUROMACS-RHF score was tested in a large population from the EUROMACS database [54]. It is composed of severe RV dysfunction, RAP/PCWP ratio ≥0.54, advanced INTERMACS classes 1–3, need for ≥3 intravenous inotropes and haemoglobin <10 g/dL. A 5-point composite score predicted early RHF after LVAD implantation, and the risk of both RHF and mortality increased as the score increased. Although the authors claimed that the EUROMACS-RHF risk model outperformed previously published scores, its AUC was good (0.75) when applied to the cohort implanted with the HeartMate II, but dropped to an unsatisfactory 0.66 for the latest-generation HeartMate III. Its limited discriminatory power was later confirmed in two external cohorts [13, 55], with AUCs of 0.64 and 0.58 respectively, casting doubt on its true clinical utility. The reasons for such different performances in different populations and devices are difficult to identify. Some hypotheses may include the tendency to better prepare patients for implantation more recently, for example by ensuring they have a higher haemoglobin concentration before surgery, the absence of preoperative RAP/PCWP measurement in patients under ECMO support, and their percentage in the whole population. Another possibility is the need for a learning curve in the management of the “new” HeartMate III, which may have impacted the occurrence of postoperative RHF.

An attempt to compare the predictive performance of each model was made by Peters *et al*. [56] who retrospectively applied different scores, decision trees and echocardiographic metrics to their cohort of 93 LVAD patients. Interestingly, the older and more established Michigan score, which emphasises preoperative haemodynamic instability and target end-organ dysfunction, remained a superior predictor of postoperative RHF as well as of short- and long-term mortality when compared to the Utah and EUROMACS models. The Michigan RHF score was also the best predictor of in-hospital mortality and long-term survival.

However, these conclusions failed to be confirmed in a more recent retrospective single-centre analysis [57], which found that the EUROMACS-RHF risk score was superior to the Michigan and CRITT models in predicting RHF. The authors took the opportunity to develop a new model based on four variables selected for the best reduced logistic model: INTERMACS level, number of inotropes used, RAP/PCWP ratio and echocardiographic RV/left ventricle diameter ratio. This model showed significant discrimination of RHF with an AUC of 0.9, probably due to the addition of a more objective parameter of RV function assessment, but further testing in different populations is needed.

The currently available risk scores and their receiver operating curves are summarised in Table 3 (Ref. [13, 29, 53, 55]).

**Table 3.** **Performance of risk scores to predict right heart failure following left ventricular device implantation**.

Score	Variables	ROC
Michigan	vasopressors use	0.73 [validation study]
	creatinine >2.3 mg/dL	0.61 [29]
	bilirubin >2 mg/dL	0.60 [53]
	aspartate aminotransferase >80 UI/dL	
Penn RVAD risk score	cardiac index	0.51 [validation study]
	RVSWi	
	severe pre-LVAD RV dysfunction	
	creatinine	
	previous cardiac surgery	
	hypotension	
UTAH	intra-aortic balloon counterpulsation	0.743 [validation study]
	increased PVR	0.52 [29]
	destination therapy	
Kormos	RAP/PCWP >0.63	0.61 [29]
	ventilatory support	0.63 [53]
	BUN >39 mg/dL	
CRITT	RAP >15 mmHg	0.67 [29]
	severe RV dysfunction	0.74 [53]
	intubation	
	severe tricuspid regurgitation	
	tachycardia	
ALMA	destination therapy	0.77 [validation study]
	PAPi <2	
	RVSWi <300 mmHg/mL/m^2^	
	RV/LV ratio >0.75	
	MELD-XI score >17	
EUROMACS-RHF	severe RV dysfunction	0.75 [validation study]
	RAP/PCWP ≥0.54	0.66 [29]
	INTERMACS classes of 1–3	0.64 [55]
	need for ≥3 intravenous inotropes	0.58 [13]
	hemoglobin <10 g/dL	
Valente	INTERMACS level	0.9 [validation study]
	number of inotropes	
	right atrial/PCWP	
	RV/LV diameters	

BUN, blood urea nitrogen; LVAD, left ventricular assist device; PVR, pulmonary vascular resistance; ROC, receiver operating curve; RVAD, right ventricular assist device.

### 5.8 Intraoperative Echography and Haemodynamic

To our knowledge, only one study has focused on the intraoperative evaluation of some haemodynamic and echocardiographic measures that could predict the development of severe RHF after LVAD implantation. Gudejko *et al*. [58], in their cohort of 110 LVAD patients, identified RAP and PAPi after chest closure as reliable factors associated with RHF, whereas quantitative echocardiographic metrics of right heart geometry and function acquired after cardiopulmonary bypass were weakly correlated. Although such considerations are quite appealing, caution must be exercised in drawing simple conclusions, since the early post-implantation period is a very unstable time, experiencing rapid and unexpected changes related to bleeding, adjustment of pump speed, degree of filling, importance of inotropic support, which can alter haemodynamic parameters independently of real RV function.

### 5.9 Contractile and Haemodynamic Reserve

Assessment of RV contractile reserve by pharmacological stress or exercise testing appears to be key in the evaluation of impaired resting RV function. For example, Guazzi *et al*. [59] measured TAPSE at rest and at peak exercise: patients with impaired RV function at rest but preserved contractile reserve had better RV-pulmonary coupling, ventilatory efficiency and functional capacity. Even in the context of screening for future LVAD support (which can be thought of as a stress test, since it restores normal left cardiac output and forces the RV to adapt to the new situation), and if the patient’s general condition allows for stress testing, the presence of improved RV contractility during exercise may predict a good RV response to LVAD support.

Remaining in the field of pre-implant pharmacological testing, the cohort of Read *et al*. [60] underwent vasodilator testing with nitroprusside during their pre-LVAD RHC. Multivariable analysis revealed that peak stroke volume index (SVI) was significantly associated with early RHF, with a 16% increased risk of early RHF per 1 mL/m^2^ decrease in SVI. In addition, follow-up of 10 consecutive patients showed that all patients were appropriately classified as having early RHF or no RHF according to this index. Moreover, resting haemodynamics demonstrated no discriminatory power.

### 5.10 Diffusion Capacity of the Lung for Carbon Monoxide (DLCO)

DLCO reduction is a common finding in HF and is associated with a worse prognosis. Its correlations with pulmonary hypertension, and in particular with higher PVR and diastolic pulmonary gradient, have been described prior to LVAD implantation, suggesting that it may be an expression of persistent lung damage in combined post- and capillary pulmonary hypertension; however, DLCO impairment failed to predict early development of RHF or significant morbidity after left support placement [61].

### 5.11 Combination of Several Predictive Factors

To corroborate their findings and compare different tools for predicting the early occurrence of RHF after LVAD support, many authors have attempted to evaluate the performance of different pre-implant features in the same cohort of patients.

Liang *et al*. [27] found that echocardiographic RV strain (AUC 0.86) outperformed more invasive conventional haemodynamic measures (including PAPi and RVSWi), as well as the Michigan RV and CRITT scores, both of which were highly specific for RHF at higher values, but were not sensitive.

These conclusions are in complete contrast to those of Peters *et al*. [56] who found that the Michigan score was superior to PAPi, preoperative RV dysfunction, RAP and PVR in predicting RHF after LVAD. In another group of LVAD patients, preoperative transpulmonary gradient, cardiac index and postoperative PAPi were the only haemodynamic variables associated with the development of RHF. However, the best predictive power was obtained when the Michigan score was combined with post-implant PAPi, with an area under the receiver operating characteristic curve of 0.73 [47].

Sert *et al*. [62] described the performance of different risk scores and their combinations (Michigan, Pennsylvania, CRITT, ALMA and EUROMACS) in a retrospective study of 71 patients who underwent continuous-flow LVAD placement between 2013 and 2016. Each model alone showed poor discrimination (AUC below 0.7), with slightly better performance for the CRITT and EUROMACS scores. The addition of the Michigan and Pennsylvania models did not improve their predictive power, while the association of TAPSE + Pennsylvania was found to have the highest sensitivity (85%), while TAPSE + Michigan + RAP/PCWP appeared to be the most specific combination.

Once again, it is clear that unanimity is far from being achieved. This may be due to the retrospective nature of the cited validation studies and their application to small populations (<100 included patients).

### 5.12 Other Factors

Several parameters have been identified as strong predictors of the development of RHF after LVAD:

(1) Patients with a small left ventricle (with a cut-off left ventricular end-diastolic diameter of 59 mm) have an increased risk of requiring temporary RV mechanical support, as well as a higher incidence of late RHF, poorer short- and medium-term survival, and more frequent readmissions due to gastrointestinal bleeding and low flow alarms [63].

(2) Pre-operative atrial fibrillation appears to show a trend (although not statistically significant) towards an association with a higher rate of early RHF after implantation [64].

(3) The role of residual mitral regurgitation after LVAD support may be important: in their cohort of 155 consecutive patients, Sharma *et al*. [65] describe an expected improvement in the severity of both tricuspid and mitral insufficiency as a result of effective left ventricular unloading in the majority, although about 15% of device recipients had persistent significant residual mitral regurgitation, which is associated with RV dysfunction and higher long-term mortality. This may be predicted by preoperative greater left ventricular end-systolic diameter, right ventricular end-diastolic diameter, left atrial volume index and ischaemic aetiology. A recent meta-analysis [66] confirmed the effect of LVAD in improving left ventricular haemodynamics and promoting resolution of mitral regurgitation. Furthermore, the authors showed that the presence of pre-deployment moderate to severe mitral insufficiency (grade II–III) did not affect mortality or morbidity, but increased the risk of developing RHF.

(4) Tricuspid regurgitation (TR) is common in patients with end-stage HF and is part of an interplay with other risk factors (e.g., RV dysfunction, pulmonary hypertension, and renal or hepatic impairment). Many questions remain unanswered, including the impact of tricuspid insufficiency after device implantation on long-term mortality and the impact of concomitant surgical correction on postoperative outcome and in particular on the development of RHF [67]; current guidelines recommend consideration of tricuspid valve surgery in the presence of moderate to severe regurgitation at baseline. However, the key seems to be appropriate patient selection, taking into account the aetiology of TR, the severity of RV dysfunction (it is important to remember that closing a retrograde exit door in an impaired RV can be detrimental) and the underlying myocardial disease. Primary TR (e.g., caused by a defibrillator or pacemaker lead) may not decrease spontaneously after LVAD placement, whereas functional TR is likely to do so. However, functional TR can be caused not only by annular dilatation but also by valve tethering, which will not be completely reduced by annuloplasty. Overall, TR decreases after device implantation regardless of pre-LVAD pulmonary hypertension or RV function. Therefore, additional tricuspid surgery may be redundant in most patients, even considering that it has been associated with an increased risk of RV dysfunction at 3-month follow-up, possibly indicating a subtle, unrecognised RV impairment in these patients [68].

(5) Concerning molecular mechanisms potentially involved, preliminary data suggest a role for SPARC-related modular calcium-binding 2 protein (SMOC2) and TRAPP6AC in favouring RHF after LVAD, although further confirmation in larger populations is needed [69].

(6) A mini-invasive approach through a limited thoracotomy appears to significantly protect against the occurrence of RHF after LVAD placement, thereby reducing the incidence of all-cause mortality, acute renal failure and stroke [70].

(7) The development and application of artificial intelligence and machine learning is a promising area, although its performance is currently far from ideal [71].

### 5.13 IABP 

IABP remains a simple and widely used mechanical assistance that can help to stabilise decompensated patients with advanced HF. Some evidence in the medical literature [72, 73] described that IABP, placed through the femoral or subclavian approach, improved some parameters reflecting RV function, allowing a lower rate of RHF development in case of LVAD support. These data were confirmed by a report from the INTERMACS registry [74] showing that patients with IABP prior to LVAD, despite having a more advanced HF stage including worse RV function, had the same outcomes compared to the less severe patients who did not require pre-LVAD IABP, suggesting a protective role of the balloon against RHF development. The physiological mechanisms underlying such a phenomenon may include an increase in oxygen delivery to the right myocardium, improvement in right coronary perfusion and, more generally, a reduction in diastolic pulmonary artery pressure and PCWP; logically, these benefits become less likely the more fibrotic the RV free wall is [75].

## 6. RHF Post-LVAD Under ECMO Support and the Role of Impella 

The small amount of certainty disappears when the clinical situation becomes more critical and the patient is on ECMO support. In this condition, the RV is unloaded and the assessment of its contractile performance becomes even more difficult. Among the possible factors associated with the development of RHF after LVAD implantation, biochemical features lose all predictive power, whereas the clinical background prior to device implantation may retain its importance, suggesting long-standing RV dysfunction or only a transient decompensation in an acute setting requiring ECMO.

Echocardiographic assessment of the RV is currently also performed in ECMO, but the problem of RV load dependence makes it less feasible. Perhaps an exclusionary reason can be considered: the presence of multiple impaired parameters describing RV function or a significant TR at maximum ECMO support is a contraindication to isolated left support. Furthermore, even if the RV appears to be functional, it is possible that after many days of complete rest, it may have difficulty regaining full contraction immediately after restoration of full left output thanks to the LVAD. Interestingly, in the cohort of Gumus [15], despite the fact that 25% of the patients were supported by ECMO at the time of speckle-tracking echocardiography, RV free wall strain and stroke work index efficiently predicted the occurrence of RHF even in this category.

Similar to echocardiography, RHC and risk scores (which are mainly based on echocardiographic and haemodynamic variables) are not applicable due to the false haemodynamic state created by ECMO.

Regarding the other pre-implantation characteristics that influence the incidence of RHF, the findings of small left ventricular size and significant mitral regurgitation may keep their importance, as they should be independent of ECMO support.

Recently, in an attempt to overcome all these difficulties, an alternative strategy for assessing RV contractile reserve on ECMO has been proposed: it is based on switching from ECMO to an Impella 5.0 or 5.5 ® surgically implanted in the axillary artery [76]. The rationale is to reproduce pure left-sided support to test the ability of the RV to generate sufficient output. If the haemodynamic situation remains stable and the RV performs well, LVAD implantation can be planned with a high degree of confidence. However, there are several prerequisites for this approach, including the absence of apical thrombi, sufficient size of the subclavian artery and a perfectly functioning lung, as Impella does not provide ventilatory support. Moreover, this approach lacks validation in a larger population. However, in our opinion, it is an appealing strategy when doubts persist and the patient’s clinical status can tolerate two major delayed interventions (first, surgical implantation of the Impella, followed by placement of the LVAD or BiVAD about fifteen days later).

## 7. Conclusions

RHF after LVAD implantation is a persistent issue despite the rapid development of technology durable mechanical circulatory support of the left ventricle. Since it is associated with a significant increase in morbidity and mortality, which becomes more and more devastating if it is not recognised preoperatively and consequently treated with a temporary rescue right device in the few hours after LVAD placement, its pre-implantation detection is of paramount importance. Unfortunately, the RV is a difficult anatomical entity to study for several reasons, including its complex geometry, its dependence on loading conditions and also the lack of consensus in defining the criteria of true RV dysfunction [77]. Furthermore, we must not forget that we are dealing with patients with end-stage HF, involving certainly the LV, but perhaps also the right one to a greater or lesser extent. The real problem is to distinguish between irreversible and severe HF and moderate HF, which is certainly present but allows sufficient adaptation to the new haemodynamic conditions after support.

In this setting, it is clear that a monomodal assessment of the RV does not provide sufficient prognostic information about its future behaviour and that a comprehensive evaluation of the patient is required, starting from his clinical status, through echocardiography and RHC, to the application of multiple risk scores.

**Fig. 1.**
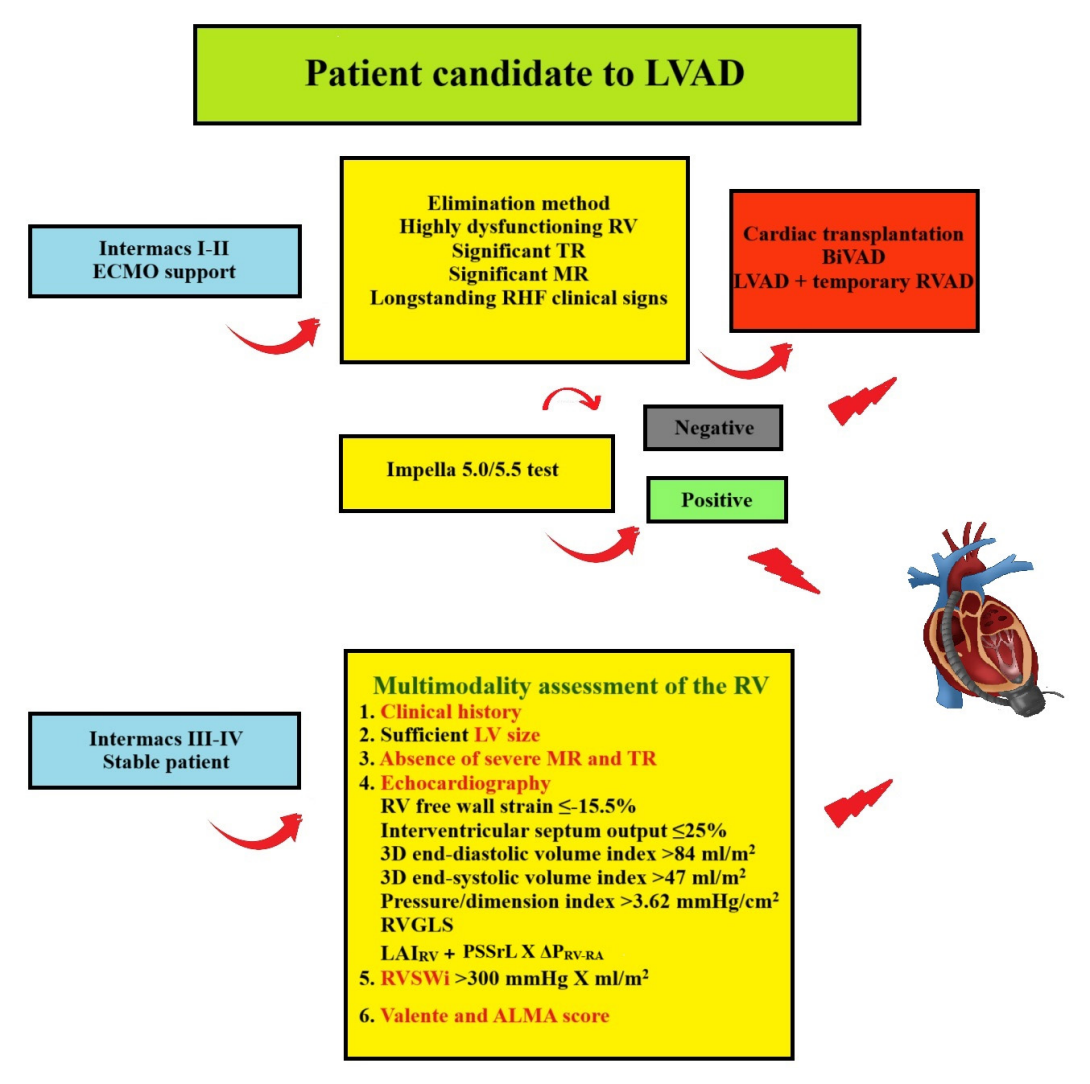
**Suggested flow chart for LVAD candidates**. Depending on the INTERMACS class, the assessment of the RV in patients screened for LVAD implantation follows a different pathway, with the aim of detecting preoperative significant RHF requiring heart transplantation or biventricular support. BiVAD, biventricular assist device; ECMO, extracorporeal membrane oxygenation; MR, mitral regurgitation; RHF, right heart failure; RVGLS, global longitudinal strain. Graphics software: Paint.

The aim of the present review is to summarise the performance of the different available tools for the assessment of RV pre-LVAD, based on the consideration that the diagnostic approach is different when the patient is in INTERMACS Class I or II on temporary support such as ECMO, or in Class III or IV (Fig. 1).

In the latter case, we are faced with a chronic stable situation in which a careful application of multiple markers may lead to an isolated left support with an acceptable risk versus a “d’emblé” biventricular assistance in the perspective of avoiding the development of high-risk RHF following LVAD implantation. More specifically, in this steady situation, the most promising and solid diagnostic variables are represented by a coexistent combination of some multimodal features, which are also quite easy to measure and apply in daily practice:

(1) Objective clinical and biochemical signs of chronic advanced RHF (such as permanent leg discolouration or persistent elevation of bilirubin).

(2) The presence of a small LV and severe pre-deployment mitral regurgitation and TR, which are particularly correlated with the development of late RHF.

(3) Several echocardiographic variables, assessed not only by basic echocardiography but also by 3D evaluation and speckle-tracking technology, including 2D and 3D free wall strain, 3D RV end-diastolic and end-systolic volumes, RVGLS, interventricular septum output, pressure/dimension index and, most importantly, LAI_RV_ and RV load-corrected peak global systolic longitudinal strain rate (PSSrL × ΔP_RV-RA_).

(4) RHC derived index represented by RVSWi.

(5) Valente risk score and, with inferior performance, the ALMA model.

Obviously, if all these parameters are favourable, isolated LVAD placement seems reasonable and safe, otherwise, if all criteria are not met, a long-term BiVAD or a planned associated temporary RV assist device can be considered in the hope of rapid recovery and adaptation of the right heart [40].

However, when patients are dependent on ECMO, the scenario changes radically as most echocardiographic, haemodynamic and biochemical values become unreliable due to complete RV unloading. Similarly, risk models based on the same measures are not applicable. In this setting, perhaps the only strategy that can be suggested is a method of exclusion: the presence of significant TR and/or a severely dysfunctional RV with complete cavity unloading under maximal ECMO support has a strong negative predictive value, discouraging single LV support. Certainly, other pre-existing factors such as a small LV, a high degree of mitral regurgitation and a clinical background suggesting longstanding RHF may argue in favour of biventricular support. In the absence of leading factors, we believe that an Impella 5.0/5.5 trial in this setting is the best option to avoid making successful LVAD placement a matter of luck.

This comprehensive and multimodal algorithm is a practical proposal based on the currently available evidence that identifies the most powerful pre-implant variables, but further validation in a large population is required.
